# Development and Evaluation of a Low-Cost Triglyceride Quantification Enzymatic Biosensor Using an Arduino-Based Microfluidic System

**DOI:** 10.3390/bios13080826

**Published:** 2023-08-17

**Authors:** Jorge E. Pliego-Sandoval, Arturo Díaz-Barbosa, Luis A. Reyes-Nava, María Angeles Camacho-Ruiz, Laura Elena Iñiguez-Muñoz, Osmar Pinto-Pérez

**Affiliations:** 1 Centro Universitario del Sur, Departamento de Ciencias Computacionales e Innovación Tecnológica, Universidad de Guadalajara, Av. Enrique Arreola Silva No. 883, Colón, Cd Guzmán 49000, Jalisco, Mexico; aalejandro.diaz@alumnos.udg.mx (A.D.-B.); luis.reyes@cusur.udg.mx (L.A.R.-N.); laural@cusur.udg.mx (L.E.I.-M.); cristofer.pinto8724@alumnos.udg.mx (O.P.-P.); 2 Centro Universitario del Norte, Laboratorio de Investigación en Biotecnología, Universidad de Guadalajara, Colotlán 46200, Jalisco, Mexico; angeles_camacho@cunorte.udg.mx

**Keywords:** enzymatic biosensor, triglycerides, lipase enzyme, microfluidic, spectrophotometric transducer, optical detector, arduino

## Abstract

Overweight and obesity promote diabetes and heart disease onset. Triglycerides are key biomarkers for cardiovascular disease, strokes, and other health issues. Scientists have devised methods and instruments for the detection of these molecules in liquid samples. In this study, an enzymatic biosensor was developed using an Arduino-based microfluidic platform, wherein a lipolytic enzyme was immobilized on an ethylene-vinyl acetate polymer through physical adsorption. This low-cost optical biosensor employed a spectrophotometric transducer and was assessed in liquid samples to indirectly detect triglycerides and fatty acids using *p*-nitrophenol as an indicator. The average triglyceride level detected in the conducted experiments was 47.727 mg/dL. The biosensor exhibited a percentage of recovery of 81.12% and a variation coefficient of 0.791%. Furthermore, the biosensor demonstrated the ability to detect triglyceride levels without the need for sample dilution, ranging from 7.6741 mg/dL to 58.835 mg/dL. This study successfully developed an efficient and affordable enzymatic biosensor prototype for triglyceride and fatty acid detection. The lipolytic enzyme immobilization on the polymer substrate provided a stable and reproducible detection system, rendering this biosensor an exciting option for the detection of these molecules.

## 1. Introduction

Triacylglycerols, commonly known as triglycerides (TGs), are composed of a glycerol backbone esterified with three fatty acids [1]. TG concentration measurement of samples has gained increased importance due to its essential role in cellular metabolism, as an energy source, and for lipoprotein formation for the transport of dietary lipids. Determining triglyceride concentration can also provide information about potential health risks associated with various diseases [2,3].

Typical TG blood levels range between 40 and 150 mg/dL; however, it is considered borderline high if the level falls between 151 and 199 mg/dL. On the other hand, a level of 200 to 499 mg/dL is classified as very high [4]. Individuals with high levels of fatty acids and TGs tend to present conditions such as obesity, lipid disorders, and insulin resistance, leading to various diseases, such as heart attacks, strokes, pancreas inflammation, and atherosclerosis [5,6,7,8]. When the level of TGs in serum exceeds 500 mg/dL, it is used as a biomarker for cardiovascular disease [9].

An alternative for measuring triglyceride levels is through the use of biosensors. This is a tool that can help in the diagnosis, monitoring, and control of diseases related to triglyceride levels in the blood, especially cardiovascular diseases [9]. Biosensors rely on several detection systems, including amperometric [10], titrimetric [11], colorimetric [12], and spectrophotometric [13] systems. However, not all detection systems are feasible to implement in constructing a biosensor due to the high cost of the materials or reagents required [14,15].

Electrochemical transducers are highly preferred for biosensor construction due to their accuracy, rapidity, and sensitivity [16]. Based on their respective designs, there are some drawbacks associated with these transducers. These include potential issues when switching sample types, a limited number of usage cycles, a relatively short half-life [7], and possible interference in the ion sample [17].

Optical transducers are an advantageous option as they exhibit remarkable applicability in the field of healthcare. They feature high sensitivity, mechanical stability, low construction costs, easy integration, and real-time detection, and they can be integrated into lab-on-a-chip systems. These characteristics make them a valuable alternative to be considered [18].

Recently, there has been a growing trend toward employing various methods to incorporate an optical transducer and collect information conveniently and efficiently. As a result, the concept of the lab-on-a-chip device has emerged, enabling easy sample injection [19]. The on-chip laboratories possess multiple features that make them ideal for triglyceride analysis. They allow for sample injection, on-chip deposition, droplet formation structures, fluidic pathways, and detection sites through reactions on the same substrate. Additionally, they reduce sample and reagent volumes, minimize waste production, lower costs, increase energy efficiency, and minimize analysis time. Immobilized metabolites are also utilized, providing a comprehensive solution [20,21,22].

Various carriers have been investigated for immobilizing enzymes to detect TGs. These carriers include pectin [13] and polyvinyl chloride [23]. Polymers, as highlighted in the studies by Reddy and Narang [23,24], are particularly useful due to their adsorption capacity, thermal stability, environmental feasibility [13], and enabling the detection of various biomolecules [22].

Lipase is a versatile enzyme widely used in various fields such as clinical, environmental, industrial, process control, and development [24]. It catalyzes the hydrolysis of carboxylic ester bonds of TGs, resulting in the production of fatty acids, acylglycerides, and glycerol. It is applied and extends to the detection of TGs [7]. Additionally, studies reveal that multienzyme extracts containing lipases, glycerol kinase, and glycerol-3-phosphate oxidase are useful for triglyceride detection [25,26].

Therefore, this work proposes the development of a new microfluidic-based system capable of integrating a biosensing system for TG quantification using a lipolytic enzyme, an optical transducer, and an Arduino data acquisition board.

Several applications have been developed concerning optical transducers and the use of Arduino boards. One of the most representative examples is the work by Nandiyanto et al. [27], where a BH1750 sensor was utilized to construct a spectrophotometer using a white LED and an Arduino electronic card as an acquisition system for analyzing curcumin concentration.

Another noteworthy application is the work by Mitra et al. [28], who developed a portable device for colorimetric quantification of ketones and glucose levels in human urine. This device also utilized the same type of sensor along with an Arduino board.

For more specific applications, Hassanah et al. [13] manufactured an optical biosensor for detecting TGs using a combination of pectin hydrogel, a pH indicator, and a lipase enzyme. Iyer and Chattopadhyay [29] developed a biosensor to detect triglyceride contaminants with a colorimetric detection and a porcine pancreatic lipase immobilized nanocomposite.

Furthermore, Weston et al. [30,31] created a colorimetric biosensor for food quality detection. Weston’s biosensor identified free fatty acids as a quality marker in almond milk, allowing for the distinction between TGs and free fatty acids. The biosensor, made of polydiacetylenes and agarose, changes color from blue to red when exposed to spoiled almond milk.

## 2. Materials and Methods

### 2.1. Reagents and Materials

Chemicals used in this research include recombinant lipase B from *Candida* sp., expressed in *Aspergillus niger*, trioctanoin, *p*-nitrophenol (*p*-NP), *p*-nitrophenyl butyrate (*p*-NPB), Bradford Reagent, and bovine serum albumin, all of which were obtained from Sigma-Aldrich^®^ (St. Louis, MI, USA).

In addition, the experimental setup included the utilization of various materials. For instance, the Ethylene-vinyl acetate (EVA) polymer or XP500 sourced from PromaPlast was employed. In addition, solvents and reagents encompassed ethanol, isopropyl alcohol (2-propanol), 3-(N-morpholino) propane sulfonic acid (MOPS), Triton X100, and phosphate buffer (50 mM, pH 7.2). All other chemicals and solvents used were of analytical reagent grade and spectroscopic grade, respectively. Furthermore, the equipment configuration featured a violet LED (light-emitting diode), a transduced BH1750 sensor, a 1.3${}^{\prime \prime }$ OLED display using I2C communication, PLA filament for 3D printing, and an Arduino micro board.

### 2.2. Instruments Used

An LED and optical transducer BH1750 were used to measure absorbance in the biosensor. The absorbance signal from the reaction mixture in the biosensor was compared with a Thermo Scientific™ Multiskan SkyHigh spectrophotometer. Additionally, essential laboratory equipment, such as an AREC magnetic stirrer and a BIORAD UltraRocker lateral stirrer, were utilized. The microfluidic device was fabricated using Ultimaker s3.

### 2.3. Enzyme Activity and Triglyceride Quantification

In order to determine enzymatic and triglyceride quantification, two different methods were employed. The first methodology was developed by Kordel et al. [32] and uses a chromogenic substrate; this method has a high level of sensitivity to confirm enzyme immobilization and transducer applicability.

However, the Kordel method does not involve the use of TGs; for that reason, the methodology of Mateos et al. [33] was implemented, which allows for the use of a triglyceride and *p*-nitrophenol as a chromophore to establish the level of fatty acids. To determine the concentrations of TGs, Glasser et al. [34] devised a method that takes into account three fatty acids released to calculate the activity. In the case of the CalB enzyme, it releases a fatty acid at the sn-1 position of the triglyceride structure [35,36], generating a diacylglycerol [37].

Furthermore, the protein concentration in the enzymatic extract was quantified using the procedure described by Bradford, which involves the use of bovine serum albumin as a standard [38]. This allowed for the estimation of the specific activity of the lipase, expressed in U/mg.

#### 2.3.1. Enzyme Activity Using *p*-NPB as a Substrate

**Figure 1:**
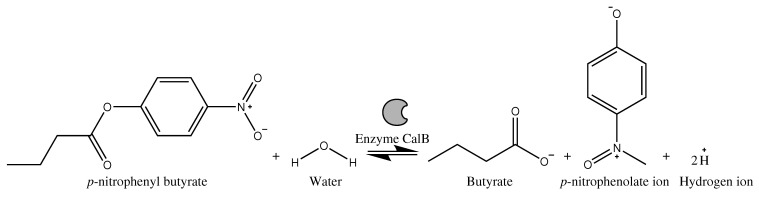
Hydrolysis reaction of the substrate *p*-NPB catalyzed by the enzyme lipase.

To determine enzymatic activity, the methodology of Kordel et al. [32] was used with some modifications. The Kordel method utilized *p*-nitrophenyl butyrate as a chromogenic substrate due to its exceptional sensitivity, quickness, and user-friendliness. For further details, please refer to Figure 1.

Initially, a stock solution of *p*−nitrophenyl butyrate (*p*-NPB) at 10 mM in 2−propanol was prepared and kept frozen until use. Additionally, a phosphate buffer solution at 50 mM, pH 7.0, was prepared.

For each well of the microplate, 180 μL of buffer (18 parts), 10 μL of substrate (one part), and 10 μL of enzyme extract or immobilized enzyme (3 mg) were mixed. The reaction was carried out at a temperature of 30 ${}^{{}^{\circ}}$C for 9 min, with 30 s intervals between each reading at a wavelength of 415 nm. Immediately afterward, the molar extinction coefficient of the released *p*−nitrophenol *p*−NP (ϵ) was measured spectrophotometrically at 415 nm using a standard curve of *p*−NP, with concentrations ranging from 0 to 500 μM. One unit (U) of activity is defined as the amount of enzyme that will convert one μmol of substrate (*p*−NP) in one minute (μmol/min) [39,40,41]. Equation (1) shows how the enzymatic activity was calculated [42]:

(1) $$\begin{array}{c} \frac{U}{mL}=\frac{\left|\Delta A\right|}{\epsilon }*\frac{\alpha }{\beta }*\gamma \end{array}$$

where:

Δ*A* = kinetic slope (Abs/min);
α = reaction volume (μL);
β = sample volume (μL);
ϵ = molar extinction coefficient (min/μmol);
γ = appropriate sample dilution.

#### 2.3.2. Enzymatic Activity Using TGs and *p*-Nitrophenol as a Reaction Indicator and Calculation Process of TG Quantification

**Figure 2:**
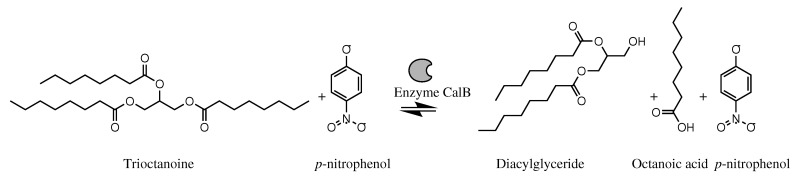
Hydrolysis reaction of trioctanoin using *p*-nitrophenol as an indicator catalyzed by the enzyme lipase.

The methodology of Mateos-Díaz et al. [33] was employed, with certain modifications; see Figure 2. A stock solution of trioctanoin at 25 mM in 2-propanol and a solution of *p*-NP 2.5 mM in 2-propanol were prepared. The buffer used was MOPS 2.5 mM pH 7.2, supplemented with 0.5% (*w*/*v*) Triton X100. The reaction mixture consisted of an emulsion containing 0.5 parts (5 μL) of triglyceride solution, 0.5 parts (5 μL) of *p*-NP solution, nine parts (90 μL) of buffer, and two parts of either enzyme extract or immobilized enzyme. A standard curve of different concentrations of octanoic acid from 0 to 10 mM in the presence of *p*-NP was measured spectrophotometrically at 415 nm under the same conditions as the reaction. One unit (U) of activity is defined as the amount of enzyme that will convert one μmol of substrate (octanoic acid) in one minute (μmol/min); see Equation (1).

After calculating the enzyme activity, it was multiplied by the duration of triglyceride hydrolysis to determine the number of moles released. Similar to the calculation of TGs, the conversion to mg/dL was carried out [43]. For the validation of the biosensor, the release of octanoic/caprylic acid was used as a standard to determine the total amount of TGs (trioctanoin) [44].

This is based on the moles of fatty acids obtained from enzymatic activity in triglyceride calculations [34,45]. According to the literature, the CalB enzyme has a preference for the sn-1 bond in the triglyceride structure [35,36], resulting in the release of a single chain of fatty acids and 2-monoacylglycerol molecules [37]. Therefore, the calculation for releasing one mole of fatty acid is based on this specificity and multiplied by three to estimate the level of TGs.

Based on the described methodology, the final TG levels in the sample can be estimated using Equation (2), where the enzyme activity reported in μmol/min is multiplied by the reaction time, the three fatty acid chains, the molecular weight of the trioctanoin, and a conversion factor to convert from (μmoles, mL, g) to (mol, dL, mg) to obtain mg/dL of the TGs in the sample.

(2) $$\begin{array}{c} TG=EA*Rt*Fac*Mw_{t}*Fc \end{array}$$

where:

TG = triglyceride concentration ($\frac{\mathrm{mg}}{\mathrm{dL}}$);
EA = enzyme activity (U);
Fac = fatty acid chains in the triglycerides (3);
$Mw_{t}$ = molecular weight of triglyceride (470.68 $\frac{g}{\mathrm{mol}}$);
Rt = reaction time (10 min);
Fc = conversion factor (0.1 $\frac{\mathrm{mL}*\mathrm{mg}*\mathrm{mol}}{\mathrm{dL}*g*\mu \mathrm{mol}}$).

### 2.4. Enzyme Immobilization with Ethylene-Vinyl Acetate (EVA) Polymer by Physical Adsorption

The methodology of Manoel et al. [46] was used as a base with some modifications, using the physical adsorption method. The EVA pellets were cut to <1 mm, and 0.5 g of the input was weighed. Subsequently, the polymer was washed with 5 mL of ethanol for 30 min under constant agitation. After that, 10 mL of water was added, and agitation was continued for 30 min. The mixture was then filtered and dried at room temperature. Following the activation of the support, 0.1 g of pellets were placed in Eppendorf tubes.

For immobilization, eight different concentrations of the lipase enzyme and phosphate buffer were used, resulting in a final volume of 1000 μL in each Eppendorf tube (Table 1). The software Statgraphics Centurion XV^®^ was utilized to improve the enzyme and polymer concentrations.

The ideal conditions for immobilization were determined through a two-way ANOVA analysis, taking into account the size of the polymer (cut or whole) and the concentration of the enzyme (ranging from 10 μL to 700 μL; see Table 1) as factors. Before conducting the ANOVA analysis, a Box–Cox transformation was executed by using the square root of the enzyme activity to obtain a normal data distribution.

### 2.5. Construction of Optical TG Biosensor and Response Measurement

Regarding the transducer, optical sensor BH1750 was used as a receiver, coupled with a violet LED and an Arduino micro board equipped with a 1.3${}^{\prime \prime }$ OLED display I2C module with a resolution of 128 × 64 and an SH1106 driver as output construction, similar to the setup described by Nandiyanto et al. [27].

Arduino code was developed, along with the corresponding electrical circuit, to trigger the emission of a light beam from the LED upon pressing a button. The sensor captures the signal transmitted through the camera and automatically calculates the necessary data for determining the concentration of TGs. To minimize light reflection, the device was coated with a dark-colored material. Prior to sampling, the device and the light intensity were calibrated using distilled water.

## 3. Results and Discussion

### 3.1. Characterization Immobilization of the CalB Lipase in EVA by Physic Adsorption

The ANOVA analysis, conducted at a 95% confidence level, did not reveal any statistically significant difference in the tested polymer sizes (*p*-value = 0.2324 > 0.05). Additionally, there was no interaction between polymer size and enzyme concentration (*p*-value = 0.7839 > 0.05), indicating that the effects of enzyme concentrations are independent of polymer size.

**Figure 3:**
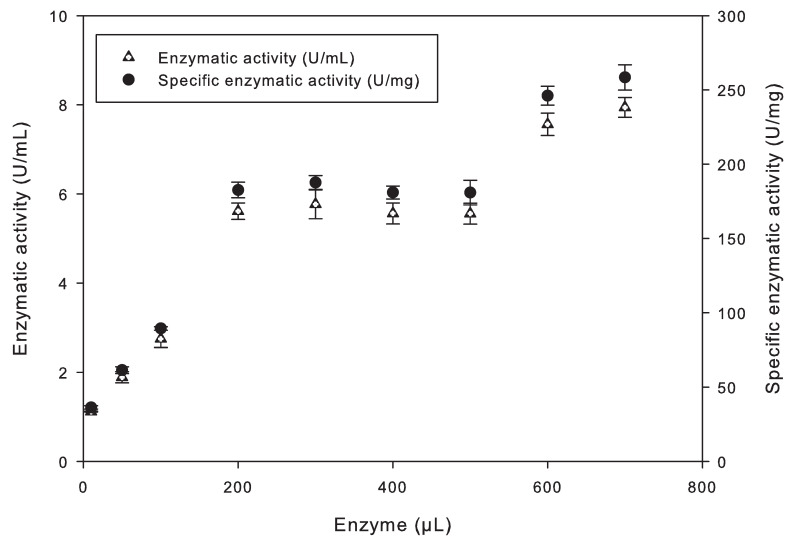
Enzymatic activities of the enzyme immobilized with EVA polymer.

On the other hand, the enzyme concentration had a statistically significant effect on the enzyme activity in the immobilized samples (*p*-value = 0.0369 < 0.05). Different amounts of the enzyme were added to the immobilized solution to enhance the process (Table 1). The graph in Figure 3 shows the correlation between the enzyme volume in the polymer and its enzyme activity (U/ml) and specific enzyme activity (U/mg). The lipase activity escalated exponentially in the initial four samples (10 μL, 50 μL, 100 μL, and 200 μL), reaching its maximum at 200 μL of the CalB enzyme, with an activity of 5.6118 U/mL.

Enzymatic activity remains within the range of 5 to 6 U/mL when the enzyme quantity ranged from 200 μL to 500 μL. Therefore, the immobilized product was selected with 200 μL of the CalB enzyme, as higher concentrations would result in unnecessary enzyme waste. It is essential to mention that, according to this immobilization optimization, 200 μL of the enzyme was used for each 100 mg of EVA, and only 9 mg to 54 mg of immobilized enzyme were placed in the biosensor.

The physicochemical properties of EVA (melting temperature, crystallinity, flexibility, adhesion, among others) vary depending on the vinyl acetate (VA) content, expanding the uses for this copolymer [47,48,49,50].

Pedersen and Eigtved [51] mentioned that the support should have a reduced pore size of no less than 100 Å. According to Cai et al. [52], the immobilization of the CalB enzyme was evaluated using different pore diameters (6.6, 8.1, and 12.5 nm), finding the highest enzymatic activity with a pore size of 12.5 nm (125 Å). Therefore, the EVA polymer, with a variable pore size ranging from 5 μm to 9 μm (depending on the %VA) [53], is considered a suitable support for the immobilization of the CalB enzyme.

### 3.2. TG Biosensor Fabrication

**Figure 4:**
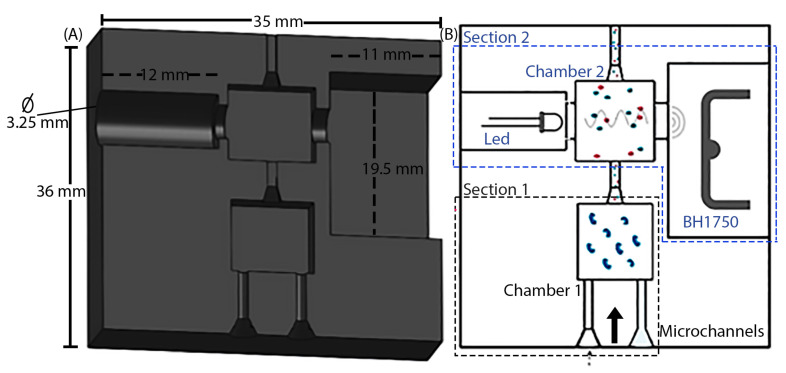
Structure of the microfluidic device for measuring the concentration of TGs. (**A**) Internal cross-section, (**B**) diagram of the structure of the biosensor.

The device has a final dimension of 35 × 36 × 17 mm in width, height, and depth (W × H × D), respectively. It consists of two interconnected sections for sample transport and analysis through channels. Figure 4A shows the 3D printed microfluidic device with a cross-section providing a view of the internal parts with detailed sections.

Figure 4B shows two sections. The first section includes a first chamber with dimensions of 8.5 × 8.5 × 5.75 mm (W × H × D) for the initial process involving the immobilized enzyme. The second section incorporates an LED light source, Gy-302 BH1750 light intensity sensor for spectrophotometric measurements, and a second chamber measuring 9 × 9 × 4.55 mm (W × H × D) to analyze the sample in a manner similar to the “stopped flow” methodology in a sequential injection analysis system [42].

The channels used for fluid transportation have a diameter of 1 mm. In the second section, two compartments are specifically designed for the light emitter and receiver. The location for the LED is cylindrical, with a radius of 3.25 mm and a width of 12 mm. Additionally, there is a circular opening of 2 mm for the passage of light into the chamber where the sample is placed. On the other hand, the BH1750 sensor section has dimensions of 11 × 19.5 × 1.5 mm (W × H × D) and includes a cylindrical opening of 2 mm positioned near the receiving sensor area.

A potentiometer was used to regulate the intensity of emitted light in the spectrophotometer. Additionally, a violet-hued light-emitting diode (LED) was employed; this is the color absorbed in the wavelength range of 390–435 nm, reflecting the greenish yellow color observed in the enzymatic reaction [54].

**Figure 5:**
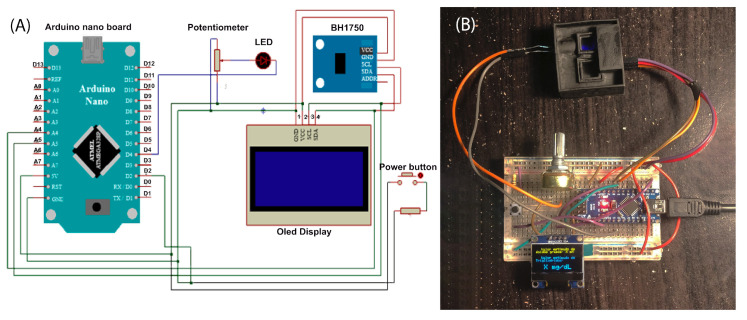
Setup and integration of enzymatic biosensor for TG quantification. (**A**) Wiring diagram of the TG biosensor device. (**B**) Enzymatic biosensor for triglyceride quantification.

A BH1750 light sensor was used to measure transmitted light. The biosensor was controlled using an Arduino Nano V3 board, programmed with Arduino software version 1.8.16. The OLED display I2C was connected as the output. Refer to the connection diagram in Figure 5A for more details. Moreover, in Figure 5B, the 3D printed device integrated with the electronic components on a breadboard can be observed.

To prevent light reflection, the device was painted in a dark color. Prior to sampling, the device and light intensity were calibrated using water as a reference substance.

It is important to acknowledge that using a low-cost sensor like the BH1750 and an LED light source may result in reduced sensitivity compared to commercial laboratory spectrophotometers, where it is possible to select a specific wavelength. The present transducer cost less than USD 1 (code 840,ISO 3166-1), and the Arduino Nano board cost less than USD 6 (code 840,ISO 3166-1), which makes this technology easily accessible for developing countries. The biosensor also has integrated microfluidic technology built on an easily accessible 3D printer and cost-effective electronics. The transducer and Arduino board have been validated in both chemical and biomedical contexts [27,28].

Commercial laboratory spectrophotometers, which were used for validation in this study, employ a xenon light source and a temperature control system, enabling measurements with high accuracy and precision, but at higher costs. In contrast, the device implemented in this work lacks a temperature control system and utilizes an LED light source, enabling measurements in a wavelength range of 390–435 nm approximately. Nonetheless, it offers the advantages of small size, portability, lower cost, and in situ monitoring.

### 3.3. Response of the TG Optical Biosensor

Once the biological and electronic components of the device were integrated, tests were carried out using the immobilized material, to compare the kinetics in the spectrophotometer with the kinetics in the biosensor.

According to the results, the biosensor exhibited an enzyme activity of 5.4176 U/mL, while the spectrophotometer measured a slightly higher activity of 6.3647 U/mL. This difference in measurements can be attributed to the sensitivity of the BH1750 sensor, which may be slightly affected at low levels of received light [55]. Additionally, the lack of a temperature control system in the device can lead to a decrease in the enzymatic kinetic reaction rate.

Based on the data provided, the Kordel method adapted to the biosensor demonstrated an accuracy rate of 85.12%, with a coefficient of variation of 1.51%.

In this study, trioctanoin was used as stock solution, with a concentration of 25 mM or 58.835 mg/dL to estimate the concentration of TGs. Dilutions were performed to avoid saturation, and the absorbance was adjusted to 1.4 at the beginning of the reaction. The enzyme’s reaction rate was measured from this point.

**Figure 6:**
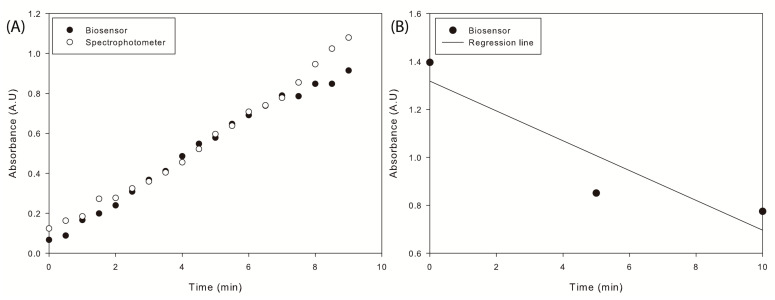
Response of substrate hydrolysis with the CalB enzyme immobilized. (**A**) Hydrolysis of *p*-nitrophenyl butyrate in a spectrophotometer and on the biosensor. (**B**) Hydrolysis of trioctanoin on the biosensor.

Based on the described methodology, the measurement of trioctanoin was performed using 54 mg of immobilized enzyme, as shown in Figure 6B. The resulting slope was calculated as 0.0622. The enzyme CalB exhibited an enzymatic activity of 0.0338 U/mL over a reaction time of 10 min. After calculating the enzyme activity, it was multiplied by the duration of triglyceride hydrolysis to determine the number of moles released. These results can be used to estimate the final TG levels in the sample based on Equation (2). Therefore, the amount of triglyceride calculated is 47.7270 mg/dL. Comparing this result with the TG standard concentration of 25 mM (1.25 μmol/mL or 58.835 mg/dL), the obtained percentage of recovery of added trioctanoin in the sample was 81.12%.

Table 2 provides a summary that compares the analytical attributes of different TG biosensors. This comparison encompasses optical, amperometric, potentiometric, impedimetric, and deoxymetric biosensors, detailing the enzyme type, immobilization method, optimal temperature, optimal pH, minimum detection limit, recovery, precision, response time, and accuracy.

Detection Limit. According to the analysis of enzyme kinetics, the maximum achievable concentration of TGs is 58.835 mg/dL without dilution. The minimum enzymatic activity is 0.005435 U/mL, which corresponds to 2.3512 mg/dL of fatty acids and 7.6741 mg/dL or 0.1630 mM of trioctanoin. The minimum detection limit is lower than that of the hydrogel pectin-based triglyceride optical biosensor (1.2387 mM; [13]) and higher than the nanocomposite biosensor (0.0113 mM; [29]). The minimum detection limit reported in some biosensors falls within the range of amperometric biosensors (10${}^{-7}$ mM to 14 mM), potentiometric biosensors (9 × 10${}^{-5}$ mM to 5 mM), and DO metric biosensors (0.0005 mM) [9].

Recovery. The percentage of recovery of added trioctanoin in samples (25 mM) in the present biosensor was 81.12% ± 0.6480% (mean ± SD; n = 5), which is comparable to the PVC membrane biosensor and the Prussian blue modified screen-printed amperometric biosensor (85.2–89.01% and 81%; [23] and [56], respectively).

Precision and response time. The within-batch coefficients of variation (CV) for TG determination in samples by the present sensor were 0.79% (n = 5), which is comparable to some colorimetric-based biosensors (2.5% and 0.0089% in [13] and [29], respectively) and lower than other electrochemical transducer biosensors (4.14% in [25]). The response time in this biosensor is 600 s, allowing for comparison with the behavior of the enzyme and the triglyceride with some colorimetric-based biosensors (300 s and 360–420 s in [13] and [29], respectively). On the other hand, certain amperometric, potentiometric, and DO metric biosensors show response times in the range or 2.5–1200 s, 30–2700 s, and 2.0–300 s, respectively; [9].

Accuracy. To study the accuracy of the present transducer, *p*-nitrophenol was measured by the Kordel method over 10 min with 30 s intervals, and the release of *p*-nitrophenolate ions by the spectrophotometer method was compared with the present biosensor. The values obtained by both methods showed a good correlation, with r${}^{2}$ = 0.9635 (Figure 6A), which is superior to the PVC membrane biosensor and the CA membrane-bound enzyme biosensor (r${}^{2}$ = 0.83 [23,57]) and comparable with the OD metric biosensor, EISCAP sensor, impedimetric/conductometric technique-based TG biosensor, and amperometric TG on pencil graphite electrode biosensor (r${}^{2}$ = 0.9409 [60], r${}^{2}$ = 0.9584 [58], r${}^{2}$=0.9801 [59], and r${}^{2}$ = 0.99 [10], respectively).

While various types of triglyceride biosensors have been developed, a novel biosensor that uses an unreported polymer to immobilize CalB enzymes was implemented, and the immobilized enzymes were tested. The present biosensor offers an economical, affordable, and accessible option for monitoring TG levels.

In this context, trioctanoin is used in medicine and research as an alternative energy source for the body and was used as a standard in the determination of TGs in this work. The consumption of trioctanoin is an alternative to moderately increasing ketones without radically altering food intake or restricting carbohydrates. The diet with these TGs has been used for many years to treat refractory childhood epilepsy [61].

It is important to measure octanoic acid, since studies have identified it as a biomarker linked to the development of colorectal cancer [62].

## 4. Conclusions

An optical biosensor for TGs was established based on the immobilized CalB enzyme and a microfluidic device. All necessary parameters for constructing this biosensor have been determined, resulting in a detection limit and a response toward TG concentration.

This biosensor is excellent for detecting TGs with acceptable accuracy and precision. The experiments demonstrate the potential of this biosensor to detect TGs. Moreover, there are plans to further develop and enhance this specific TG biosensor to optimize its performance and customize it for use in natural blood serum. The objective is to improve its functionality and ensure its compatibility for practical applications in clinical settings.

## Figures and Tables

**Table 1:** The CalB enzyme and buffer concentrations for immobilization in EVA.

Sample	1	2	3	4	5	6	7	8	9
Enzyme (μL)	10	50	100	200	300	400	500	600	700
Buffer (μL)	990	950	900	800	700	600	500	400	300

**Table 2:** Comparison of analytic characteristics of various TG biosensors.

Type of Biosensor	Biosensor Characteristics	Enzyme	Method of Immobilization	Optimum Temp (${}^{{}^{\circ}}$C)	Optimum pH	Detection Limit (mM)	Recovery (%)	Precision (%)	Response Time (s)	Accuracy	Reference
Optical	**Present biosensor**	CalB	Adsorption	30	7.2	0.1630	81.12	0.791	600	0.9635	—
Optical	Hydrogel pectin	Lip	Entrapment and Adsorption	NR	7	1.2387	NR	2.5	300	0.9807	[13]
Optical	Colorimetric Lip immobilized nanocomposite	PLip	Adsorption	25–55	6–9	0.0113	70–50	0.0089	360–420	NR	[29]
Amperometric	Prussian blue modified screen-printed	Gd, No, and Lip	Cross-linking	25–65	7.4	NR	81–99	NR	300-1800	0.9988	[56]
Amperometric	PVC membrane biosensor	Lip, Gk, and G-3-PO	Adsorption	35	7	0.28	85.2–89.01	NR	10	0.83	[23]
Amperometric	Lip/Nanoporous gold/Glassy carbon electrode	Lip, Gk. and G-3-PO	Covalent Binding	35	6.5	0.23	91–95	4.14 and 5.85	4	0.9801	[25]
Amperometric	Cellulose acetate membrane biosensor	Lip, Gk, and G-3-PO	Adsorption	25	6.5	0.2	89	<8	40	0.83	[57]
Amperometric	Pencil graphite electrode biosensor	Lip, Gk, and G-3-PO	Adsorption	35	7	10${}^{-7}$	98.01	0.05	2.5	0.99	[10]
Potentiometric	EISCAP sensor	Lip	NR	25	7.4	0.1	NR	NR	1800	0.9584	[58]
Impedimetric	Impedimetric/ conductometric	Lip	Covalent Binding	NR	NR	0.28	NR	NR	20	0.9801	[59]
Deoxymetric	Membrane-bound lipase, Gk, and G-3-PO	Lip, Gk, and G-3-PO	NR	39.5	7.5	0.35	NR	<2.18	10–15	0.9409	[60]

Lipase (Lip); Glycerol kinase (Gk); Glycerol-3-phosphate oxidase (G-3-PO); Glycerol dehydrogenase (Gd); NADH oxidase (No); Porcine pancreatic lipase (PLip); *Candida antarctica* lipase B (CalB).; Not reported (NR).

## Data Availability

Not applicable.

## Abbreviations

The following abbreviations are used in this manuscript:

TGs	triglycerides
TG	triglyceride
EVA	ethylene-vinyl acetate
PLA	polylactic acid
CalB	*Candida antarctica* lipase B
*p*-NPB	*p*-nitrophenol butyrate
*p*-NP	*p*-nitrophenol
MOPS	3-(N-morpholino) propane sulfonic acid
ANOVA	analysis of variance
VA	vinyl acetate
LED	light-emitting diode
